# Fragments from the Note-book of an Office Pupil of the Late Dr. George M’Clellan, Containing Cases and Clinical Remarks*The manuscript book from which these fragments are extracted was only preserved for its write’s own use, but the Editor has thought that records, however slight, of the views and practice of so eminent a man, would not be unacceptable to the readers of the Examiner.

**Published:** 1850-12

**Authors:** 


### MEDICAL EXAMINER,

AND

### RECORD OF MEDICAL SCIENCE.

NEW SERIES.—NO. LXXII.—DECEMBER, 1850.

### ORIGINAL COMMUNICATIONS.

*Fragments from the note-book of an office pupil of the late* Dr. George M'Clellan, *containing cases and clinical remarks.**

*May 12th*, 1843. *Calomel—effects of large doses.*—Dr. M'Clellan in referring to-day to the effects of large doses of calomel upon the system, mentioned some experiments which were made upon dogs in India by Annesley. He gave ʒj. or more of calomel to one dog, and a few grains of the same to another, and killed them both after a certain number of hours. After repeating these experiments, he invariably found that the large doses had the effect of blanching the gastric mucous membrane, contracting the intestinal exhalents, stimulating greatly the liver, and apparently of forming with the intestinal mucus, a thick saponaceous compound; while the examination of the animals treated with the small doses, developed only the engorgement of the mucous membranes which is usual after death.

*The manuscript book from which these fragments are extracted was only preserved for its writer's own use, but the Editor has thought that records, however slight, of the views and practice of so eminent a man, would not be unacceptable to the readers of the Examiner.

In remarking upon these experiments, Dr. M'Clellan said that he thought the happiest effects had resulted from doses of calomel ranging from 20 to 60 grains, in many cases where smaller quantities were usually given, as in certain forms of gastric irritation, inflammation and congestion, congestive hepatic disorder, dysentery, and the symptomatic affections of the abdominal viscera, accompanied by foul watery evacuations which frequently occur after bad wounds. Such doses generally cause only one or two evacuations, which are often more consistent than those which preceded them, stimulate largely the liver, correct the disposition to diseased exhalation, and, in general gastro-intestinal affections, often put a stop at once to vomiting and purging. He has seldom seen large doses of calomel productive of any but a pleasant effect when given in a state of system accompanied by dry skin, and generally defective secretions, and is always careful to diminish or discontinue the doses upon the re-establishment of these.

He has sometimes found that the long-continued action of calomel even in small doses, when it did not produce salivation, caused very disagreeable consequences, among the most troublesome of which was *amaurosis*, which he has occasionally seen to be produced. Mercurials given with the effect of causing slight ptyalism, he never found to give rise to such or any other unfortunate results.

*Nitrate of Silver.*—This powerful escharotic has a most delightfully mild and tranquillising effect upon the mucous membranes, and is an invaluable remedy for inflammation or irritation of those tissues. Its effects in sore throat and ophthalmia have long been known. In the latter disease it deadens the irritability, blanches the mucous membrane, contracts the vessels, and sometimes in the most painful, strumous ophthalmia, with intolerance of light, after one or two applications, enables the child to bear the full glare of the sun. He generally has it dropped into the eye in the proportions of from gr. j. to gr. iij. to a f.℥j of water, or else applies to the surface, moistened bibulous paper, previously smeared over a stick of caustic, or where granulations exist, applies the solid nitrate itself. He has found the sedative and counter-irritatant effects of the caustic freely applied to the *outside* of the lids, to assist very much in the treatment of scrofulous ophthalmia with great photophobia, as well as in the severe inflammations occurring in adults.

Dr. M'Clellan has found this remedy serviceable in allaying irritation and inflammation of other organs. He administered it in the only real case of idiopathic gastritis he ever saw, where the patient could retain absolutely nothing upon her stomach, and was afflicted with the most obstinate and constant singultus-like vomiting. After all ordinary means had failed, he gave the nitrate of silver in doses of ¼ grain, repeated as often as they were rejected. After a few hours the vomiting ceased, and she was soon, with that and other treatment, enabled to retain any kind of nourishment.

Dr. M'Clellan also finds this remedy invaluable in erysipelas; not only to set up a circumscribing barrier, but to deaden the irritability of the part, and reduce the inflammation, smearing it lightly over the skin, so as not more than to blacken it. It lessens irritability of the skin generally, can often be applied with great benefit over parts affected with *dermalgia* and *neuralgia,* and has also a delightful effect in removing the irritability of *corns* when painful. Blistering the surface with caustic over the course of a nerve affected with neuralgia, has frequently acted like a charm in his hands.

*June 19th,* 1843. *Cataract commencing.*—A young man presented himself, who has had within the last few months, an increasing dimness of sight; but which, as yet, by no means amounts to a total loss of vision. There are slight milky opacities in both his lenses, and he has rather an undue determination of blood to the eyes; iris and conjunctiva too highly colored.

The Dr. thought, that as it was only the commencing stage, it would be possible to make it disappear by the use of cathartics and slight alteratives. He therefore gave:

℞. Ext. colocynth. compos. ℈j.

Hydrarg. chlorid. mit. ʒss.

Ant. et Potass. Tart. gr. ij.

M. et divid. in pill xxx.

3 pills to be taken every 3d night.

He believes that it is often possible to stop the progress of cataract in young people, but seldom or never *in old.*

*Diseases of the Prostate Gland.*—Inflammatory enlargement of the prostate rarely occurs but in young men, and is then generally owing to irritation from gonorrhœa or similar causes. In this disease, a burning pain is felt in the part, with which the rectum sympathises so much, that the pain is often attributed to that organ. The tumor can often be perceived in perineo, but is always felt distinctly by the finger introduced into the rectum. It can be best combated by general depletion, leeches and cups to the perineum—cold and soothing applications constantly injected into the rectum, purgatives, &c.

The chronic enlargement that occurs in old men, is a very perverse disease, and often impossible to cure. Frequently the only thing that can be done, is to prevent obliteration of the urethra by the use of bougies.

The Dr. out of many cases, only knows two which have been fully reduced, and in these suppuration took place, and the *tumors were entirely removed.*

*Spontaneous Absorption of the Prostate.*—He mentioned a very interesting case of a man under the care of Dr. Morton, who, when young had been a victim to masturbation, and in after life was always subject to emissions; in coition, semen would be discharged before he had obtained an entrance. Almost lost the use of lower limbs, &c.

In him, the prostate had been entirely absorbed. By introducing a catheter and putting the finger into the rectum, it could be run along the whole course of the catheter without meeting with the prostate. He since saw a case in the dissecting rooms where there was no prostate, and where the patient appeared to be of the above description.

*Stricture from Enlarged Prostate.*—When the prostate is enlarged, the Lobulus Morgagnii, or third lobe, very often projects up across the opening, forming what may then be well called, the *palate* of the bladder or uvula vesicæ. In such cases, it is next to impossible to get a common catheter into the bladder. It must be longer than common, and *very* much curved—when with tact, it is easily passed in.

*A few remarks upon Strictures of the Urethra.*—These should at first be always examined with a wax bougie—inasmuch as by the careful use of it, the exact nature and appearance of the stricture can be easily ascertained. The wax becomes warmed in the passage, and takes an exact mould of the contracted part. If it will not enter the stricture, the application of force will expand and bend the end of the bougie, and will show the mouth of the stricture upon its point. If it will enter, the part which it has entered will be an exact reversed copy of the stricture, (dilated somewhat by pressure, to be sure,) while the force applied will bend the part behind—back in the healthy urethra.

These bougies are also very convenient for cauterising the urethra; a *small* piece of caustic must be firmly imbedded in the end of the bougie, and part of it covered over—thus there is no danger of its falling out, and any part of the stricture may be touched by it.

Patients are sometimes under treatment for months, from the idea that they have strictures, caused by the apparent impossibility of passing up a catheter. These difficulties often arise from the lacunæ or follicles of the urethra, or the prostatic ducts being enlarged, and may occur even from their existence in the natural state.

A pointed bougie will frequently catch against these obstructions and give the idea of an impassable stricture. If a large bougie with a blunt round extremity is used, it will pass the obstacle in nine cases out of ten.

It is well to carry the point of the bougie pressed upon the lower or posterior surface of the urethra, till it arrives at the pubis, for the lacunæ of the urethra are chiefly upon the anterior surface, and when it passes that part, to press it upon the anterior portion; for the verumontanum, and the prostatic and antiprostatic ducts, are upon the posterior part. To show the possibility of the catheter getting into one of these ducts, Dr. M'Clellan mentioned a case in which a surgeon had made numerous attempts to get into the bladder, in a case of prostatic enlargement. At last he changed the position of the instrument slightly. The man cried out that something had given way, and the instrument passed its whole length without difficulty, apparently into the bladder. No urine came, however, and the attempt was not renewed. The man ultimately recovered, but died a short time after of a bilious fever. On an examination it was found that the catheter had entered the sinus pocularis, gone through the ejaculatory duct, torn through the vesicula seminalis, and some recto-vesical attachments, and produced a large abscess there.

He mentioned another very interesting case in which the catheter penetrated the *ureter* by being carried too far.

It was that of a man who had swallowed a date stone, which lodged in the appendix cæci vermiformis, producing inflammation, which ended favorably in a large abscess circumscribed by the peritoneum in the lower part of the abdomen, so that the bladder floated in it. This produced a tumor above the pubis, which was mistaken very naturally for retention. Dr. Physick introduced a catheter two or three times, but only a tablespoonful or so of urine would come off. Thinking that he had not penetrated the bladder, he, on one occasion, made use of more force than usual, but with the same results. Finally the man was tapped above the pubis at Physick's request, by M'Clellan, when the state of affairs above described was found; no *urine* in the bladder, but a large abcess in front of it. The man died in a few days, when an examination developed that the catheter had passed up the ureter about an inch, tearing the mucous membranes, so that a large blister of urine had formed between the mucous and muscular coats.

*Irritable Urethra.*—Where the urethra is very irritable, the injection of a solution of nitrate of silver is an invaluable resort. In many cases of stricture, and in particular predispositions, there is great irritability of this canal, increased by the slightest touch of a bougie.

He recollects the cases of two gentlemen, brothers, suffering from stricture, in which the most gentle application of the bougie gave rise to intense agony, nervous chills, and even convulsions. In both these cases, the use of an injection of nitrate of silver, gradually increased from the strength of three grs. to an f.℥j. water up to six or ten grs. entirely cured this morbid sensibility, and in a few weeks the bougie could be introduced in both cases without any unpleasant results.

*Sept. 24th,* 1843. *Hydrocele of the Neck.**—A young gentleman, æt. 19, from Mount Sterling, Kentucky, has had a tumor in his neck, which has been increasing for two years, until it deformed him very much, presenting a uniform, œdematous-looking enlargement of all the front and sides, looking somewhat like the leg of one affected with elephantiasis. It was of a soft consistence, yielding a semiliquid feel, and had given him little pain or uneasiness, except that it produced difficulty in breathing at night, and snoring.

Dr. M'C. pronounced it to be a hydrocele of the neck, or watery tumor contained in a sac, probably encircling the thyroid gland. He determined, as the only safe course, to make an exploratory puncture, and let out the matter.

Last Sunday he did so, by introducing a lancet at the most projecting part of the swelling, upon the left side, and let out about a pint of dark bilious looking serum, tinged with blood. He injected a little warm water to clean out a few flakes that were in it, and closed up the wound, merely applying some lint upon it.

Monday morning. The tumor had filled up to its usual size, appeared to be a little inflamed; it was very difficult for him to swallow, and he was suffering from considerable constitutional derangement. He had had a slight chill in the morning, and then (10 o'clock) his pulse was quick (120) and irritated, and he was querulous and excited. Dr. M'Clellan bled him 10 ounces (he had been purged on Saturday and Sunday,) and ordered

℞. Sulph. morph., grs. iss.
Ant. and potass. tart., gr. i.
Aq. fluvial, f.℥ij.

S. 40 drops every few hours, and also gave aq. camphoræ every few hours, which remedies had the effect of calming him very much.

* This case is reported at length, with remarks uopn this affection, in M'Clellan's Surgery, page 318.

Tuesday, Wednesday, Thursday and Friday, he continued getting rather worse; at times violently delirious, and at others apparently in a typhoid state; a little subsultus tendinum and muttering delirium.

He could swallow water with difficulty, and could take no nourishment but a little liquid preparation of arrow root and ice cream, &c. Cold lotions were kept applied to the part, and on Wednesday fifty leeches were applied, which had the effect of diminishing the pain.

On Saturday the Dr. felt an obscure fluctuation on the opposite side, and opened it with a lancet, letting out f.℥j or ij. of a bloody serum, of a suspicious looking character, and he then ordered the application of poultices. From all the symptoms, he feared very much the development of fungus, a result which has often taken place in similar tumours, although, sometimes, puncture and even the employment of setons in these cases, have given rise to suppuration and obliteration of the sac, and have resulted in perfect cures.

Tuesday, Sept. 26.—His bad symptoms have all abated, and the wound is now in a state of full suppuration. He is easy and perfectly *compos mentis.* Pulse weak but natural; and there is every prospect that the process of suppuration now going on will result in the closing of the sac and spontaneous cure of the disease, without the formation of fungus.

Saturday, Sept. 30th.—The puncture on the other side, which had closed entirely, again opened yesterday, and full suppuration is now going on at the orifice, and the right and left sides now appear continuous.

Nov. 1st.—He has gone home, perfectly cured, the tumor reduced in size, there being only a little lump on the left side near the orifice of the wound, which is still suppurating freely.

